# Illness Severity, Social and Cognitive Ability, and EEG Analysis of Ten Patients with Rett Syndrome Treated with Mecasermin (Recombinant Human IGF-1)

**DOI:** 10.1155/2016/5073078

**Published:** 2016-01-26

**Authors:** Giorgio Pini, Laura Congiu, Alberto Benincasa, Pietro DiMarco, Stefania Bigoni, Adam H. Dyer, Niall Mortimer, Andrea Della-Chiesa, Sean O'Leary, Rachel McNamara, Kevin J. Mitchell, Michael Gill, Daniela Tropea

**Affiliations:** ^1^Tuscany Rett Center, Ospedale Versilia, 55043 Lido di Camaiore, Italy; ^2^School of Medicine, Trinity College Dublin, College Green, Dublin 2, Ireland; ^3^Department of Genetics, Trinity College Dublin, College Green, Dublin 2, Ireland; ^4^Trinity College Institute of Neuroscience, College Green, Dublin 2, Ireland; ^5^Department of Psychiatry, Trinity College Dublin, College Green, Dublin 2, Ireland; ^6^Neuropsychiatric Genetics, Trinity Centre for Health Sciences, St. James Hospital, Dublin 8, Ireland

## Abstract

Rett Syndrome (RTT) is a severe neurodevelopmental disorder characterized by an apparently normal development followed by an arrest and subsequent regression of cognitive and psychomotor abilities. At present, RTT has no definitive cure and the treatment of RTT represents a largely unmet clinical need. Following partial elucidation of the underlying neurobiology of RTT, a new treatment has been proposed, Mecasermin (recombinant human Insulin-Like Growth Factor 1), which, in addition to impressive evidence from preclinical murine models of RTT, has demonstrated safety in human studies of patients with RTT. The present clinical study examines the disease severity as assessed by clinicians (International Scoring System: ISS), social and cognitive ability assessed by two blinded, independent observers (RSS: Rett Severity Score), and changes in brain activity (EEG) parameters of ten patients with classic RTT and ten untreated patients matched for age and clinical severity. Significant improvement in both the ISS (*p* = 0.0106) and RSS (*p* = 0.0274) was found in patients treated with IGF1 in comparison to untreated patients. Analysis of the novel RSS also suggests that patients treated with IGF1 have a greater endurance to social and cognitive testing. The present clinical study adds significant preliminary evidence for the use of IGF-1 in the treatment of RTT and other disorders of the autism spectrum.

## 1. Introduction

Rett Syndrome (RTT) is a devastating neurodevelopmental disorder affecting approximately 1 in 10,000 females. RTT is characterized by an apparently normal period of development followed by a stagnation regression of cognitive and psychomotor abilities. Over 85% of cases are caused by mutations in the gene coding for methyl CpG-binding protein 2 (*MeCP2*) and following this discovery significant neurobiological insight has followed [1]. Moreover, a novel treatment based on such insight, IGF-1 (Insulin-Like Growth Factor 1), has shown promise in preclinical animal models [2, 3] and demonstrated safety in multiple early human studies [4–6].

Patients with RTT have no evident symptoms during the first 18 months of life. When disease becomes clinically apparent, the following symptoms appear: stagnation (cessation of growth, microcephaly, muscle loss, and hypotonia), developmental regression (deficits in social interaction, hand wringing, and clapping), decrease in BMI and musculoskeletal disorders (weight loss, osteopaenia, and scoliosis), and motor deterioration (Parkinsonian symptoms, weakness). Some authors describe the above symptoms as appearing at different stages of the disease [7]. Cardiorespiratory problems also complicate the clinical picture of RTT, contributing to the increased rates of sudden death, as does the high prevalence of epilepsy in RTT patients [8, 9].

Mutations in the* MeCP2* gene on Xq28 are responsible for RTT in the vast majority of cases [10]. The protein product of* MeCP2*, methyl-CpG-binding protein 2 (MECP2), is primarily found in the nucleus, where it binds symmetrically methylated CpG sites [11]. Also located outside the nucleus, it may act as a crucial link between synaptic activity and neuronal transcription [12, 13]. Functions of MECP2 may include transcriptional regulation and involvement in processes such as brain development, neuronal structure, and synaptic function [12]. MECP2 is critical to synaptic maturation and maintenance [14].

Importantly,* MeCP2* deletion in murine models results in a RTT-like phenotype which recapitulates many of the features of RTT in humans and is ameliorated on restoration of* MeCP2* expression. Intuitively, restoration of* MeCP2* function represents an attractive mechanism to target in the treatment of RTT [15–17]. Approaches to restore MECP2 function by gene therapy, whilst attractive, may be somewhat limited at present [18]. A more pragmatic therapeutic approach may be to target the downstream effectors of MECP2.

The best characterized downstream effector of MECP2 is Brain Derived Neurotrophic Factor (BDNF) critical in processes such as neuronal and synaptic maturation and development [19]. BDNF is downregulated in mouse models of RTT as well as in RTT patients [3, 20, 21]. Importantly, overexpression of BDNF can counteract the RTT phenotype in murine models [20]. However, the pharmacological use of BDNF is complicated by the fact that BDNF is unable to cross the Blood Brain Barrier (BBB) [22]. Another pleiotropic growth factor which has critical roles in synapse development, maturation, and maintenance is Insulin-Like Growth Factor 1 (IGF1), an activator of tyrosine kinase receptors and canonical pathways such as PI3K and MAPK signaling pathways [23, 24]. The ability of IGF-1 and its tripeptide form (GPE: Glutamate-Proline-Glycine) to cross the BBB significantly improves its potential as a therapeutic agent [11]. IGF1 pathway activation in* Mecp2* knockout mice has been shown to reverse many features of the RTT phenotype, such as anxiety levels, respiratory patterns, and irregularity in heart rate [2, 3]. The effects of IGF1 extend to increased synaptic signaling proteins, enhanced cortical excitatory transmission, and restored dendritic spine densities and amplitudes [2, 3]. Thus, IGF1 signifies a plausible therapeutic target in the treatment of RTT.

Recombinant human IGF1 (rhIGF-1, Mecasermin) is presently indicated in the pediatric population for severe growth failure and IGF1 deficiency [25]. In RTT patients, three studies to date have examined IGF-1 safety and tolerability [4–6]. The first assessed the safety of IGF1 in six RTT patients with a subsequent single case study reporting its safety in repeated cycles of IGF1 treatment [4, 5]. Further, a recent study confirmed the safety of IGF1 treatment and examined the pharmacokinetics of a multiple ascending dose and open-label extension study in 9 patients with RTT [6]. Preliminary efficacy analysis in this study noted improvements in certain cardiorespiratory measures (apnea), anxiety, and depression (indexed by reversal of frontal band alpha asymmetry on EEG).

The present clinical study investigates the changes in clinical severity (ISS) following IGF-1 treatment in ten RTT patients treated with IGF1 in comparison to untreated patients matched for clinical severity, age, and study duration. We also investigated the social and cognitive abilities of these patients in comparison to untreated patients using a novel video-based analysis, the Rett Severity Score (RSS). EEG analysis was performed following precedent in the literature for the RTT population. Although the present study is open-labeled in nature, it represents the first report in the literature on social and cognitive parameters in IGF-1 treated patients and IGF1 effects on endurance, adding significant weight to the preliminary evidence base for IGF-1 treatment in RTT patients.

## 2. Materials and Methods

### 2.1. Patient Screening and Selection

The procedures were approved by Comitato Etico Locale Sperimentazioni (CELS) Viareggio Delibera number 328 del 28/10/2009. Of twenty-five girls screened using the inclusion criteria, ten were selected for treatment with IGF1 [4]. A list of inclusion/exclusion criteria is reported in [4]. In brief: all patients had classic RTT with mutations in the* MeCP2* gene. Exclusion criteria included being aged <2 or >13 years, not in the third-stage of the disorder (according the stage-subdivision of the disease [7]), displaying nonclassical variants of RTT, presence of neoplasia, or a medical history of hypoglycaemia. Younger patients included in the study had demonstrated the regression of acquired abilities such as language and showed manual dyspraxia, all symptoms allowing an early diagnosis. Also, they demonstrated an improvement of interactive behavior, typical of a “transitory autism.”

All ten treated patients (subjects 1–10; S1–10) had International Scoring System (ISS) score assessment before (T0) and after treatment (T1). Nine of the ten treated patients had social and cognitive ability assessment at T0 and T1 by two independent blinded assessors. All had adequate EEG recordings available at T0 and T1.

Ten untreated RTT patients who were selected on matching for age and disease severity (untreated subjects 1–10; U1–10) were also included. Nine of these untreated patients had ISS ascertained at comparable time periods to treated patients (24 weeks apart at T0 and T1), with all having social and cognitive ability assessed at T0 and T1 and nine having adequate EEG available at T0 and T1. Details of the individual patients can be found in Table 1.

### 2.2. IGF1 Administration

Families gave written consent for participation in the study prior to commencing IGF1 treatment. IGF1 (Mecasermin-Increlex^*∗*^) was administered subcutaneously twice/day for 20–24 weeks to all ten patients. For the first and last week of the study, 0.05 mg/Kg was administered. For the intervening weeks, a dose of 0.1 mg/Kg was administered. The dose used is identical to that for other pediatric indications. The safety of IGF1 at this dose and similar doses in this population has been demonstrated [4–6].

### 2.3. Subgroup Analysis

#### 2.3.1. Mutation Severity Status of RTT Patients

Despite its monogenic classification, there is significant genetic heterogeneity in RTT. Stratification of RTT patients by mutation severity may indicate a variability in the benefit of IGF1 which correlates with varying degrees of mutation severity. There were eight unique mutations in the IGF-1 treated group of patients, with two individuals carrying the T158M mutation. Within the untreated group all individuals had a unique mutation. This is detailed in Table 1. Mutation severity was grouped into 3 discrete groups in accordance with previous classification in the literature [26].

### 2.4. Outcomes/Parameters Measured

#### 2.4.1. International Scoring System (ISS)

The International Scoring System (ISS) [27, 28] was used to evaluate the severity of disease in each of the patients, with a lower score indicating a lower disease severity. ISS considers scores on 5 separate subscales: (i) Growth and Development, (ii) Muscular-Skeletal Appearance, (iii) Movement, (iv) Mental-Cortical, and (v) Brainstem-Autonomic. Each item is scored as follows: 0: no abnormality, 1: mild abnormality, and 2: severe abnormality. At baseline, ISS was compared between IGF1 treated and untreated groups to assess the similarity of the groups and hence applicability of the present study to the RTT population. Following this, the change in ISS score of the ten treated patients at T0 and T1 was compared to that of the RTT untreated patients at a comparable time interval (T0 and T1).

#### 2.4.2. Social and Cognitive Analysis

In order to assess social and cognitive parameters, video footage of both treated and untreated groups was analysed by two blinded, independent observers. The observers had no prior exposure or knowledge of the treatment status of the patients. 60 minutes minimum of video footage was analysed at T0 and T1. Nine of the ten treated patients had video footage of sufficient quality available and all ten untreated patients had adequate footage available. In the remaining treated patient, adequate video footage was unavailable. Three of the subjects were evaluated only by one observer.

Preliminarily footage was observed in order to design the scoring criteria design the novel tool, the Rett Severity Scale (RSS). Patients were scored on 20 features in total (Table 2): (i) 10 positive features: pointing, manipulating, reaching for something, ability to mimic/imitate, indicating yes/no with a head gesture, reactivity to a call, reactivity to an object, smiling in response to a stimulus, deliberate vocalisation, and attention, and (ii) 10 negative features: hand wringing, biting, rocking, hitting, indiscriminate moaning, tongue chewing, vacant starting, bruxism, breath-holding apnea, and Valsalva maneuver. To assure that the scale selected was appropriate and would not lead to false positive or negative results, analysis of scores was performed twice, using an initial scoring system followed by a recoded scoring system.

In the first system each of these features was rated on a scale of 1 to 5 by severity with the following system: 1: extreme impairment, 2: severe impairment, 3: moderate impairment, 4: mild impairment, and 5: absence of impairment. The second score system, based on the average of raters initial assessment, is in the range of 1 (severe impairment), 2 (medium impairment), 3 (no impairment). In order to ensure interrater reliability and validity of the novel RSS, the correlation between both rater scores was calculated for both positive and negative symptoms at T0 and T1. For this correlation, we used the scoring system 1–5 as it is the one which more stringently considers input from independent observers. The tool is included in the supplementary materials (see Supplementary Material available online at http://dx.doi.org/10.1155/2016/5073078) of this paper as is a detailed description of each severity rating for each of the 20 features.

In addition, observers rated scores for different time points during each visit (T0 and T1). This enabled comparison of scores at the beginning and end of each visit and was carried out in order to enable analysis of testing endurance in IGF1 treated and untreated patients. Importantly, all girls who had video footage recorded had it recorded in the same room, for the same period of time with the same level of external stimulation, noise, and activity. Such a methodology has clear benefits in terms of assessment standardization.

#### 2.4.3. EEG Analysis

All ten treated patients and nine untreated patients had EEG measurements carried out using ten electrodes EEG at the same time as video footage (above) was recorded. Electrodes were positioned in accordance with the 10–20 layouts and lasted 1-2 hours. Analysis was performed for a minimum duration of 28 minutes each, sampled at a frequency of 128 kHz (samples/sec). Analysis was conducted using a custom-made script in MATLAB consisting of a Fast Fourier Transform (FFT) function which analyses the signal divided in five second bins. Peak and mean frequencies and amplitudes for the different ranges were separately averaged over the whole signal and grouped into bins to be used as comparative variables between and within groups [4]. Following precedent literature on EEG findings in RTT, mean theta and delta wave amplitude and frequencies and alpha band power were examined [4, 6, 9, 29].

### 2.5. Statistical Analysis

Due to the nature of data used and the small sample size in the present study, nonparametric tests were used. The Mann-Whitney *U* test was used for unpaired analysis and the Wilcoxon signed rank test for paired analysis. A Kruskal-Wallis test was used to mutation severity subgroups. For parametric data, paired and unpaired *t*-tests were used. For inferential analysis, tests used were two-tailed, with the threshold for statistical significance being set at *p* < 0.05. Statistical analysis was performed using Minitab 17 and GraphPad Prism 6.0, with the latter also being used to create figures of the relevant results.

## 3. Results

### 3.1. Significant Improvement in ISS Scores in IGF-1 Treated RTT Patients

The International Scoring System (ISS) was used to assess RTT severity as detailed above. There was no difference in ISS ratings between treated and untreated groups at baseline (*p* = 0.967, Mann-Whitney *U* test), reflecting the clinical similarity of the treated and untreated RTT patients.

**Figure 1:**
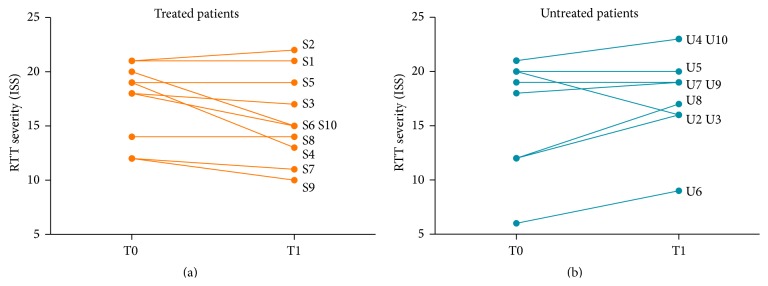
ISS before and after treatment for (a) IGF1 treated and (b) untreated patients. The Mean change in ISS in IGF1 treated versus untreated patients (*p* = 0.0106, Mann-Whitney *U* test, two-tailed). The change in ISS for the treated group was short of significance (*p* = 0.052, Wilcoxon Signed Ranks Test, two-tailed). ISS: International Scoring System. S1–10: treated patients 1–10; U2–10: untreated patients 1–10.

In the treated group, six patients showed an improvement (decrease) in ISS with three patients showing no change. One of the treated patients (S2) had an ISS increased by one point at follow-up. In the untreated group, six patients showed an increase in ISS, with two patients demonstrating no change and one patient showing an improvement in ISS. The mean change (T1–T0) in ISS was significant in the treated versus untreated patients (*p* = 0.0106, Mann-Whitney *U* test) (Figure 1). On paired analysis of treated patients, the change in ISS as a result was just short of significance (*p* = 0.052, Wilcoxon Signed Ranks Test), possibly resulting from the small sample size and the nonparametric nature of the statistics used. In sum, the change in ISS was significantly different between treated and untreated RTT patients, with the vast majority (nine from ten) of treated RTT patients showing an improvement or stability in disease severity.

### 3.2. Social and Cognitive Abilities

In order to measure the ability of patients in interacting with objects and people in the environment, we introduced a new evaluation score: the Rett Severity Score (RSS), which is described in detail in the methods and supplementary sessions. Two independent observers, blinded to the patient condition and to the time point scored the footages of treated and untreated patients. To assure the reliability of this new scoring system, the outcomes were analysed according to two different scoring systems: one ranking 1–5 and the other 1–3.

#### 3.2.1. Blinded Observers Have Correlated Evaluations' Scores

In order to assess the interrater reliability of the Rett Severity Score (RSS), the consistency between the two blinded assessors was analysed. The change in RSS ratings between each visit for each patient was calculated (T1–T0). Using a nonparametric Spearman correlation for random seven treated patients and six untreated patients, there was significant correlation between assessors ratings for positive, negative, and combined features in both treated (*p* = 0.008, *p* = 0.014, and *p* < 0.005) and untreated (*p* = 0.042, *p* = 0.015, and *p* < 0.015) groups.

#### 3.2.2. Significant Improvement in Social and Cognitive Ability in IGF1 Treated Patients

In order to appreciate RSS changes between T0 and T1, the similarity of groups at baseline was analysed. There was no difference in the average social and cognitive ratings between treated and untreated groups for positive, negative, or combined features between treatment groups.

**Figure 2:**
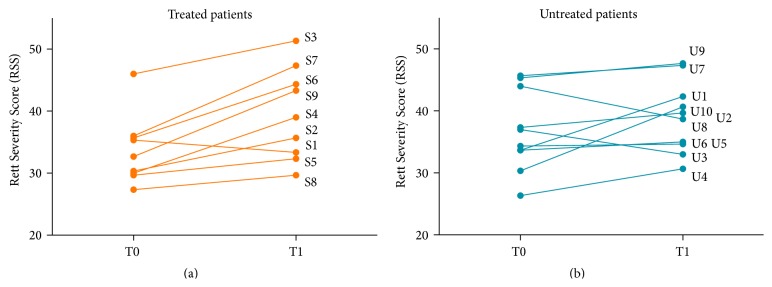
Combined features of social and cognitive scores before and after treatment for IGF1 treated patients in comparison to untreated patients. The change in Rett Severity Score (RSS) was significant for RSS 1–3 (*p* = 0.0078) as analysed by Wilcoxon Matched-Pairs Signed Ranks Test. A higher score correlates to better social/cognitive ability.

In order to compare RSS changes before and after treatment, the mean change in RSS (T1–T0) ratings by the two observers was taken. On paired analysis, the improvement in IGF1 treated patients from T0 to T1 was significant with both scoring systems (*p* = 0.009 (score: 1–5), *p* = 0.008 (score: 1–3), Wilcoxon Signed Ranks Test) but not for untreated RTT patients illustrating that significance cannot be fully accounted for by improvement with repeated testing. Thus, the patients treated with IGF1 demonstrated a significant improvement in social and cognitive scores between T0 and T1 in comparison to untreated patients (Figure 2).

Considering between-group analysis, there was a significant level of improvement in the total RSS of IGF1 treated patients in comparison to untreated patients measured with the 1–5 score system (*p* = 0.0274, Mann-Whitney *U* test). Improvement in negative features (*p* = 0.0498, Mann-Whitney *U* test) was significant in the treatment group in comparison to untreated patients, with positive features falling just short of significance (*p* = 0.061, Mann-Whitney *U* test). The same analysis with 1–3 score system just fails to reach significance (*p* = 0.056) probably for the limited number of patients in the study and the nonparametric test used for the analysis.

#### 3.2.3. Improvement in Endurance in IGF1 Treated Patients

**Figure 3:**
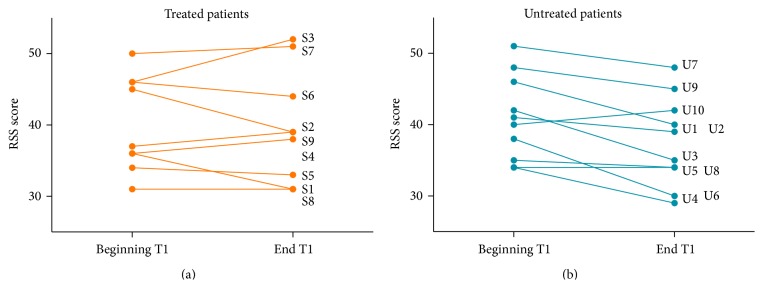
IGF1 treated patients do not show the same decrease in endurance to testing as untreated RTT patients. Endurance refers to the decrease in social/cognitive rating (RSS) from the beginning to the end of each visit (T0 and T1). In untreated patients, there is a significant decrease in scores from the start to the end of testing at T1. In treated patients, such a decrease was not seen following IGF-1 treatment. Recall that an increasing (positive on the graph above) score indicates improvement in social and cognitive ability.

As detailed in the Methods section above, social and cognitive features were rated over the course of both T0 and T1. In order to examine endurance to testing in both groups, the RSS at the beginning and end of each visit was compared. In assessing both groups of patients (*n* = 19) at baseline (T0) before IGF1 treatment, all patients showed a significant decrease in cognitive and social abilities from the beginning to the end of the assessment (*p* = 0.003 (score: 1–5) *p* = 0.021 (score: 1–3), Wilcoxon Signed Ranks Test). In assessing untreated patients on the second visit (T1), there was also a significant decrease in social and cognitive abilities from the beginning to the end of testing at T1 in positive and negative features combined (*p* = 0.014 (score: 1–5), *p* = 0.016 (score: 1–3)). Thus, repeated testing did not seem to significantly alter endurance to testing in untreated patients at T1. In IGF1 treated patients, there was no trend of increased or decreased scoring as testing progressed for both features combined (*p* = 1) or individually for negative (*p* = 0.678) or positive (*p* = 0.374) features at T1 with both scoring ranges. Thus, IGF1 may prevent the decrease in social and cognitive testing endurance in RTT patients seen over visit duration (Figure 3).

### 3.3. EEG Analysis 

#### 3.3.1. Analysis of Alpha Band Desynchronisation

Following previous reports in the RTT literature changes in frontal alpha band asymmetry in the ten RTT patients treated with IGF-1 in comparison to the ten untreated patients were investigated. Left > Right (*L* > *R*) activation is interpreted as more anxious and Right > Left (*R* > *L*) as less anxious. At T0, six patients in the treatment group had *L* > *R* desynchronisation index and four had a *R* > *L* desynchronisation index.

Overall, the change in alpha band desynchronisation in the treated group from T0 to T1 was not significant in the ten patients treated with IGF1 in comparison to the nine untreated patients (*p* = 0.8, unpaired *t*-test). Similarly, the change from T0 to T1 wasn not significant when performing paired analysis (*p* = 0.69, paired *t*-test). However, when analysis was limited to only those patients who had a *L* > *R* desynchronisation at baseline, there was a trend towards reversal on paired analysis at T0 (*p* = 0.21, paired *t*-test). The change was much weaker on unpaired analysis with the four patients in the untreated group with *L* > *R* index at baseline (*p* = 0.8196, unpaired *t*-test). When the same analysis was repeated only on the first five minutes of EEG analysis at T0 and T1, the change in alpha band desynchronisation index was not significant (*p* = 0.68 and *p* = 0.64, unpaired *t*-test and paired *t*-test).

#### 3.3.2. Delta and Theta Amplitudes and Frequencies

**Figure 4:**
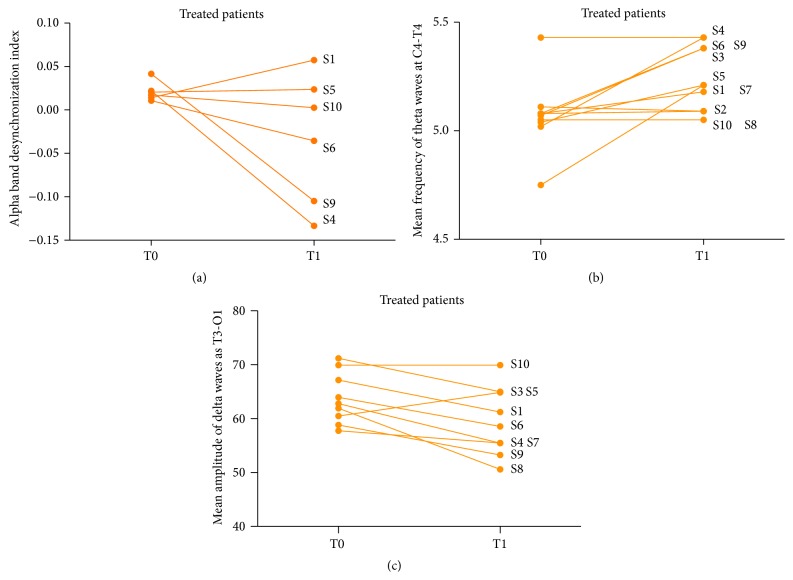
EEG parameters assessed before (T0) and after (T1) IGF-1 treatment. Alpha band desynchronisation is shown for those patients who were more anxious at baseline *L* > *R* (a). The mean frequency of theta waves at C4-T4 (b) is also shown as is the mean amplitude of delta waves at T3-O1 (c). S1–10: treated subjects 1–10.

Delta and theta amplitude and frequencies were examined. Both paired and unpaired analyses were performed examining changes in the ten IGF1 treated RTT patients in comparison to the nine untreated RTT patients. Whilst a significant signal was seen for significant changes in the mean frequency of theta waves at C4-T4 (*p* = 0.015, paired *t*-test, Figure 4), as well as for the mean amplitude of delta waves at T3-O1 (*p* = 0.019, paired *t*-test, Figure 4), the results do not survive Bonferroni correction for multiple testing. No significant results were found on unpaired analysis.

## 4. Discussion

The present clinical study examined disease severity, social cognitive ability, and brain activity (EEG) measures of ten girls with classic RTT treated with Mecasermin (rh-IGF1) in comparison to ten untreated girls. Significant improvement was found when comparing disease severity and social and cognitive abilities of patients treated with IGF-1 for 6 months before (T0) and after (T1) treatment to the untreated patients at two similar time points 6 months apart (T0/T1). Analysis of brain activity (EEG) demonstrated a trend towards a reversal of alpha band desynchronisation in IGF-1 treated patients; however the result did not reach statistical significance. Importantly, the present study represents the first empirical report into the social and cognitive abilities of RTT patients treated with IGF-1 using the novel Rett Severity Score (RSS), a novel scoring tool for blinded assessors. This analysis gives some insights into the possible effects of IGF1 in RTT, in particular when considering the increased endurance to testing seen in IGF-1 treated patients. These findings add significant preliminary evidence for the use of IGF-1 in the treatment of RTT.

Notwithstanding the open-label nature of the present study, we present the development of a novel scoring tool (the RSS) in the video-based assessment of social and cognitive abilities in RTT. This analysis, based on ratings by two blinded and independent assessors, gives the first indication to date of the possible effects of IGF-1 treatment on the social and cognitive abilities of patients with RTT. In fact, it can be suggested from the current study that part of the effect of IGF-1 may be due to an increased ability to endure assessment. Such a proposal is corroborated by anecdotal evidence from patient's clinicians and families reported with IGF-1. Although the major effect of IGF1 treatment can be appreciated only on the improvement of negative symptoms, whilst improvements of positive symptoms fail to reach significance, the number of subjects and the nonparametric nature of statistics used may have contributed to this outcome. Of note, the significant change in social and cognitive abilities as well as endurance to testing is seen using both scoring systems. This novel RSS can be considered a complementary assessment of social and cognitive ability to clinical evaluation of RTT subjects. It would be useful to examine the RSS in formal efficacy studies to confirm our preliminary results.

The International Scoring System (ISS), a clinician based assessment, is widely accepted in the assessment of disease severity in RTT. In addition to analysis of social and cognitive features, the ISS includes parameters for the assessment of movement and musculoskeletal function. All treated patients and all untreated patients except for one in the present study had their ISS assessed at T0 and T1. A note here on potential bias is prudent. Due to the open-label nature of the present study, clinicians were aware of which patients were receiving treatment with IGF-1 and which patients were not. However, notwithstanding such a limitation, all patients in the treatment group, except for one, experienced an improvement or stabilisation in ISS from T0 to T1. This is in stark contrast to the untreated group of patients, where only one patient's ISS improved from T0 to T1. The mean change in ISS from T0 to T1 was significant on both unpaired (Mann-Whitney, *p* = 0.0106) and paired (Wilcoxon, *p* = 0.009) analysis.

An important paper has reported reversal of alpha band desynchronisation on treatment with IGF-1 [6]. In the first instance, we were not able to replicate such result. However, when limited to patients with *L* > *R* alpha band desynchronisation at T0 (more anxious), paired analysis demonstrated a trend to reversal in the previously reported direction (*p* = 0.21, paired *t*-test). Such a result may not reach statistical significance due to the small sample size of IGF-1 treated patients with *L* > *R* desynchronisation at T0 (*n* = 6). Further EEG analysis in larger studies of IGF-1 treatment in RTT may help add clarity to this issue, which is complicated by the small sample sizes in the RTT population.

When analysing theta and delta waves, thought to be important in the RTT population for their role in cognition and learning, a significant change in the mean frequency of theta waves at C4-T4 (*p* = 0.015, paired *t*-test) and mean amplitude of delta waves at T3-O1 (*p* = 0.019, paired *t*-test) was demonstrated. Such results may be complex to interpret, as they do not survive testing for multiple correction and may represent false positives. The fact that the results cannot pass Bonferroni correction, as well as the fact that they cannot be seen on unpaired analysis, complicates the interpretation. It is important that future studies on IGF-1 treatment in RTT revisit such associations to enable further clarification.

A limitation of the present report is the open-label nature of the study. Whilst the ISS was not fully blinded, the RSS was assessed by blinded assessors. Notably, the strongest results in the present study are seen for the RSS. When interpreting the results of this study a key aspect is the sample size, which is a common factor of rare diseases and in this case is also affected by limited availability of IGF1; hence this study should be considered a presentation of clinical cases rather than a clinical trial. Several statistical tests fail to reach significance because of the limited sample size and the statistic test used; hence the replication of study results by additional clinical studies and trials is particularly important. In sum, the present study adds significant preliminary evidence for the use of IGF-1 in the treatment of RTT.

## 5. Conclusions

The present study demonstrated significant improvement in clinical severity and social and cognitive scores of patients with classical RTT before and after IGF-1 treatment in comparison to untreated patients matched for age and disease severity assessed at a similar interval. The present study is the first report in the literature to examine the social and cognitive abilities of RTT patients before and after treatment using the blinded RSS. Further, analysis by subgroups and analysis of various EEG parameters, whilst not meeting the threshold for statistical significance, provide preliminary evidence for parameters and variables that may be examined in the interpretation of formal efficacy data when it becomes available from clinical trials. In addition, this is first study showing that IGF1 improves endurance of treated patients, hence giving insights into the possible mechanisms of action of the treatment. The present report adds significant preliminary evidence for the use of IGF-1 in the treatment of Rett Syndrome and other disorders of the autism spectrum.

## Supplementary Material

Supplementary Information: we report the detailed criteria for the marking of the Rett Severity Score (RSS): a new evaluation score designed to capture and measure the social and cognitive abilities of Rett Patients.

## Figures and Tables

**Table 1:** Patients included in the present study.

Patient	Age	Epilepsy (Y/N)	Mutation	ISS before study
IGF-1 treated subjects				
S1	4.7	Y	R270X (nonsense)	19
S2	4.8	Y	Del exons 3 and 4	21
S3	5.11	Y	R133C (missense)	18
S4	10.8	N	1155 del 12 + 1157 del 44 (frameshift)	20
S5	8.1	Y	T158M (missense)	21
S6	5.9	Y	C13ins in GCCGC in exon 1 (frameshift)	19
S7	9.49	Y	R306C	12
S8	4.46	N	T158M (missense)	14
S9	3.72	N	1096 del 89	12
S10	3.92	Y	1157 del46n	18

Untreated subjects				
U1	2.19	N	C468G (missense)	—
U2	6.74	Y	P152R (missense)	17
U3	2.41	N	753 del C (frameshift)	12
U4	4.61	Y	R294X (nonsense)	16
U5	4.7	N	R270X (nonsense)	20
U6	12.06	Y	Y141X (nonsense)	7
U7	1.92	N	R106W (missense)	15
U8	2.66	Y	1085 del 183 (frameshift)	15
U9	3.09	N	R168X (nonsense)	18
U10	4.96	Y	T158M (missense)	21

Details of ten subjects treated with IGF-1 and ten untreated subjects included for analysis in the present study. Subjects treated with IGF-1 are numbered S1–S10 and untreated subjects are named U1–10. Age is included in years. Epilepsy status at any time point in the present study is included as is the mecp2 mutation in each individual subject. The ISS (International Scoring System) before treatment is also included, except in U1 where it was not available.

**Table 2:** Positive and negative features of the novel Rett Severity Score (RSS).

Rett Severity Score features	
Positive features	Negative features
Pointing	Hand wringing
Manipulating	Biting
Reaching for something	Rocking
Ability to mimic/imitate	Hitting
Indicating yes/no with head gesture	Indiscriminate moaning
Reactivity to a call	Tongue, chewing
Reactivity to an object	Vacant staring
Siming to stimulus	Bruxism
Deliberate Vocalisation	Breath holding, apnoea
Attention	Valsalva Maneuver

A full description can be found in the supplementary materials of the present paper.
