# Supplementary material for: Illness Severity, Social and Cognitive Ability, and EEG Analysis of Ten Patients with Rett Syndrome Treated with Mecasermin (Recombinant Human IGF-1)
Source: Autism Res Treat. 2016 Jan 26;2016:5073078. doi: 10.1155/2016/5073078 (PMC4746298; doi:10.1155/2016/5073078)
Supplement: Supplementary file 1 — Supplementary Information: we report the detailed criteria for the marking of the Rett Severity Score (RSS): a new evaluation score designed to capture and measure the social and cognitive abilities of Rett Patients. [file 5073078.f1.pdf]

**Supplementary Information**

Below we report the detailed criteria for the marking of the Rett Severity Score (RSS): a new evaluation score designed to capture and measure the social and cognitive abilities of Rett Patients.

**Scale 1-5**

**Hand Wringing**

1. Subject consistently moves hands in an uncontrollable and repeated manner. Subject is unable to stop bringing hands together repeatedly and stereotypically. The motion severely interferes with the subject's ability to manipulate objects and gesture appropriately.
2. Subject consistently moves hands together in an uncontrollable and repeated manner. Subject is unable to stop bringing hands together repeatedly and stereotypically. The motion moderately interferes with subject's ability to manipulate objects and gesture appropriately.
3. Subject often moves hands together in an uncontrollable and repeated manner. Subject is able to prevent stereotypical hand movements to some extent, especially when her attention is elsewhere. The motion interferes with the subject's ability to manipulate objects and gesture appropriately to some degree.
4. Subject sometimes moves hands together in an uncontrollable and repeated manner. Subject has little trouble preventing stereotypical hand movements when her attention is held elsewhere. The motion does not interfere with the subject's ability to manipulate objects and gesture appropriately.
5. Subject does not consistently move hands in an uncontrollable and repeated manner.

**Biting**

1. Subject consistently bites, or attempts to bite, her hands, objects around her, or her lips in an uncontrollable and repeated manner. Subject is unable to stop biting objects in her immediate surroundings.
2. Subject consistently bites, or attempts to bite, her hands, objects around her, or her lips in an uncontrollable and repeated manner. Subject is unable to stop biting objects in her immediate surroundings, except when her attention is held elsewhere.
3. Subject often bites, or attempts to bite, her hands, objects around her, or her lips in an uncontrollable and repeated manner. Subject is able to stop biting objects in her immediate surroundings when her attention is held elsewhere.
4. Subject occasionally bites, or attempts to bite, her hands, objects around her, or her lips in an uncontrollable and repeated manner. Subject stops biting objects in her immediate surroundings when her attention is held elsewhere.
5. Subject does not consistently bites, or attempts to bite, her hands, objects around her, or her lips in an uncontrollable and repeated manner.

**Rocking**

1. Subject consistently and repeatedly rocks violently back and forth in an uncontrollable manner. Subject is unable to stop the violent rocking motion. Rocking motion may involve the head, or the entire body.

2. Subject consistently and repeatedly rocks violently back and forth in an uncontrollable manner. Subject is unable to stop the violent rocking motion except when her attention is held elsewhere. Rocking motion may involve the head, or the entire body.
3. Subject consistently and repeatedly rocks back and forth in an uncontrollable manner. Subject is able to stop the rocking motion when her attention is held elsewhere. Rocking motion may involve the head, or the entire body.
4. Subject occasionally rocks back and forth in an uncontrollable manner. Subject stops the rocking motion when her attention is held elsewhere. Rocking motion may involve the head, or the entire body.
5. Subject does not rock back and forth, except in a manner which would be appropriate for a child.

**Superficial Breathing**

1. Subject consistently takes loud, rapid and shallow breaths. Subject appears to constantly be in mild respiratory distress, and is unable to stop rapid, panting breathing.
2. Subject consistently takes loud, rapid and shallow breaths. Subject appears to be in mild respiratory distress, and is unable to stop rapid, panting breathing.
3. Subject often takes loud, rapid and shallow breaths. Subject appears to be in mild respiratory distress, and is unable to stop rapid, panting breathing except when her attention is held elsewhere.
4. Subject sometimes takes loud, rapid and shallow breaths. Subject does not appear to be in respiratory distress and is unable to stop rapid, panting breathing when her attention is held elsewhere.
5. Subject does not take loud, shallow breaths.

**Moaning/Shouting**

1. Subject consistently vocalises loudly and without intent. Subject appears in great distress, and cries, shouts or moans indiscriminately.
2. Subject often vocalises loudly and without intent. Subject appears in moderate distress, and cries, shouts or moans indiscriminately.
3. Subject sometimes vocalises loudly and without intent. Subject appears in mild distress, and cries, shouts or moans indiscriminately.
4. Subject sometimes vocalises loudly and without intent. Subject does not appear to be in distress, but occasionally cries, shouts or moans indiscriminately.
5. Subject does not vocalise loudly and without intent.

**Tongue Chewing**

1. Subject consistently and repeatedly chews her tongue in an uncontrollable and stereotypical manner. Action interferes with feeding or vocalising.
2. Subject consistently and repeatedly chews her tongue in an uncontrollable and stereotypical manner. Subject is able to stop tongue chewing when feeding or vocalising.
3. Subject often repeatedly chews her tongue in an uncontrollable and stereotypical manner. Subject is able to stop tongue chewing when feeding or vocalising.

4. Subject occasionally chews her tongue in an uncontrollable and stereotypical manner. Subject is able to stop tongue chewing when feeding or vocalising.
5. Subject does not chew her tongue in an uncontrollable and stereotypical manner.

**Vacant Staring**

1. Subject shows no interest in her surroundings. Subject constantly stares at nothing in particular and pays no attention to any visual stimuli.
2. Subject shows very little interest in her surroundings. Subject constantly stares at nothing in particular and pays very little attention to any visual stimuli.
3. Subject shows little interest in her surroundings. Subject often stares at nothing in particular and pays little attention to any visual stimuli.
4. Subject shows some interest in her surroundings. Subject often stares at nothing in particular but pays some attention to visual stimuli.
5. Subject shows interest in her surrounding and responds appropriately to visual stimuli.

**Bruxism**

1. Subject constantly and consistently grinds her teeth and moves her jaw in a stereotypical and repeated manner. Action interferes with feeding and is highly associated with “biting” and “moaning”.
2. Subject constantly and consistently grinds her teeth and moves her jaw in a stereotypical and repeated manner. Action may interfere with feeding and is associated with “biting” and “moaning”.
3. Subject often grinds her teeth and moves her jaw in a stereotypical and repeated manner. Action does not interfere with feeding and is associated with “biting” and “moaning”.
4. Subject occasionally grinds her teeth and moves her jaw in a stereotypical and repeated manner. Action does not interfere with feeding and is not associated with “biting” or “moaning”.
5. Subject does not grind her teeth to any extent.

**Breathholding/Apnea**

1. Subject goes through constant cycles of holding her breath for prolonged periods of time. Highly associate with “superficial breathing” and “valsalva manouver”. Subject appears in moderate respiratory distress.
2. Subject goes through constant cycles of holding her breath for moderate periods of time. Highly associate with “superficial breathing” and “valsalva manouver”. Subject appears in mild respiratory distress.
3. Subject goes through occasional cycles of holding her breath for moderate periods of time. Highly associate with “superficial breathing” and “valsalva manouver”. Subject does not appear in respiratory distress.
4. Subject sometimes holds her breath for moderate periods of time. Subject does not appear in respiratory distress.
5. Subject does not hold her breath or stop breathing for any significant amount of time.

**Valsalva Manouver**

1. Subject constantly holds her breath and then forcibly expels it in a stereotypical manner. Subject is unable to stop performing the valsalva manouver and appears to be in moderate distress.
2. Subject consistently holds her breath and then forcibly expels it in a stereotypical manner. Subject is unable to stop performing the valsalva manouver and appears to be in mild distress.
3. Subject often holds her breath and then forcibly expels it in a stereotypical manner. Subject has difficulty stopping performing the valsalva manouver and appears to be in mild distress.
4. Subject sometimes holds her breath and then forcibly expels it in a stereotypical manner. Subject does not have difficulty stopping performing the valsalva manouver and does not appear to be in distress.
5. Subject does not hold he breath and forcibly expel it in a stereotypical manner.

**Pointing**

1. Subject is completely unable to gesture toward any stimuli of interest to her using her body or hands.
2. Subject has extreme difficulty gesturing toward any stimuli of interest to her using her body or hands.
3. Subject is able, on occasion, to indicate toward any stimuli of interest to her using her body or hands.
4. Subject consistently and repeatedly attempts or succeeds in gesturing toward a stimulus of interest using her body or hands.
5. Subject effectively points using her fingers or hands at stimuli of interest.

**Manipulating**

1. Subject is completely incapable of holding or turning any object of interest or of feeding herself.
2. Subject can barely hold objects of interest and is unable to feed herself without aid.
3. Subject can hold objects without aid but is unable to turn them around or operate them appropriately in any way.
4. Subject can feed herself relatively easily and can turn and play with objects of interest.
5. Subject can feed herself without aid, and can play with objects of interest in an appropriate manner using her hands.

**Reaching for something**

1. Subject is completely incapable of reaching for objects of interest or of indicating her interest in these objects in any way. Highly associated with “pointing” and “manipulating”
2. Subject can indicate by gesture that she wishes to hold an object but is completely incapable of reaching for or holding said object.

3. Subject shows limited ability to reach for and hold objects of interest.
4. Subject can reach for and hold objects of interest to some degree.
5. Subject can reach for and hold objects of interest with relative ease.

**Waving Goodbye**

1. Subject is unaware or completely incapable of using hand gestures to communicate goodbye, or anything else, to any other person.
2. Subject is aware of but incapable of using hand gestures to communicate goodbye, or anything else, to any other person.
3. Subject attempts to use hand gestures to communicate goodbye, or anything else, to any other person.
4. Subject attempts and occasionally succeeds in using hands gestures to communicate goodbye, or anything else, to any other person.
5. Subject tries to and consistently succeeds in using hand gestures to wave goodbye or communicate in any other way with other persons.

**Yes/No with head movement**

1. Subject is unaware or completely incapable of using head gestures to communicate yes or no, to any other person.
2. Subject is aware of but incapable of using head gestures to communicate yes or no, to any other person.
3. Subject attempts to use head gestures to communicate yes or no, to any other person.
4. Subject attempts and occasionally succeeds in using head gestures to communicate yes or no, to any other person.
5. Subject tries to and consistently succeeds in using head gestures to communicate yes or no to other persons.

**Reactivity to a Call**

1. Subject displays no outward indication of attention on being called by another person.
2. Subject displays extremely poor outward indication of attention on being called by another person.
3. Subject turns toward a call made by another person, but has no other reaction apart from this.
4. Subject turns and reacts to the person calling, to some extent.
5. Subject has no trouble turning toward and communicating in a limited sense with the person who has called them.

**Smiling in response to a stimulus**

1. Subject does not react to any stimulus of interest (Eg, a video/toy).
2. Subject cries or gives some other inappropriate response to a stimulus of interest.
3. Subject smiles in response to a stimulus of interest but inappropriately, at the wrong time, toward the wrong stimulus or other.
4. Subject smiles and laughs appropriately toward a stimulus of interest to some extent.
5. Subject has no difficulty smiling and laughing to a stimulus of interest in an appropriate manner.

**Deliberate Vocalisation**

1. Subject does not vocalise apart from indiscriminate moans and cries.
2. Subject rarely cries and laughs at appropriate times toward appropriate stimuli.
3. Subject indicates by deliberate vocalisation her emotions toward a particular stimulus.
4. Subject attempts to use her vocalisations to communicate to some degree with another person.
5. Subject is able to use deliberate vocalisations such as half words, laughs, and cries etc, to communicate to a greater degree than in "4" with another person.

**Attention**

1. Subject shows no interest in her surroundings. Subject constantly stares at nothing in particular and pays no attention to any stimuli.
2. Subject shows very little interest in her surroundings. Subject constantly stares at nothing in particular and pays very little attention to any stimuli.
3. Subject shows little interest in her surroundings. Subject sometimes pays limited attention to a stimulus of interest.
4. Subject shows interest in her surroundings. Subject pays attention to a stimulus of interest.
5. Subject shows great interest in her surroundings.

**Scale 1-3**

**Hand Wringing**

1. Subject consistently moves hands in an uncontrollable and repeated manner. Subject is unable to stop bringing hands together repeatedly and stereotypically. The motion severely or moderately interferes with the subject's ability to manipulate objects and gesture appropriately.
2. Subject often moves hands together in an uncontrollable and repeated manner. Subject is able to prevent stereotypical hand movements to some extent, especially when her attention is elsewhere. The motion interferes with the subject's ability to manipulate objects and gesture appropriately to some degree.
3. Subject rarely moves hands together in an uncontrollable and repeated manner. Subject has little trouble preventing stereotypical hand movements when her attention is held elsewhere. The motion does not interfere with the subject's ability to manipulate objects and gesture appropriately.

**Biting**

1. Subject consistently bites, or attempts to bite, her hands, objects around her, or her lips in an uncontrollable and repeated manner. Subject is unable to stop biting objects in her immediate surroundings, except when her attention is held elsewhere.
2. Subject often bites, or attempts to bite, her hands, objects around her, or her lips in an uncontrollable and repeated manner. Subject is able to stop biting objects in her immediate surroundings when her attention is held elsewhere.
3. Subject occasionally bites, or attempts to bite, her hands, objects around her, or her lips in an uncontrollable and repeated manner. Subject stops biting objects in her immediate surroundings when her attention is held elsewhere.

**Rocking**

1. Subject consistently and repeatedly rocks violently back and forth in an uncontrollable manner. Subject is unable to stop the violent rocking motion except when her attention is held elsewhere. Rocking motion may involve the head, or the entire body.
2. Subject consistently and repeatedly rocks back and forth in an uncontrollable manner. Subject is able to stop the rocking motion when her attention is held elsewhere. Rocking motion may involve the head, or the entire body.
3. Subject occasionally rocks back and forth in an uncontrollable manner. Subject stops the rocking motion when her attention is held elsewhere. Rocking motion may involve the head, or the entire body.

**Superficial Breathing**

1. Subject consistently takes loud, rapid and shallow breaths. Subject appears to constantly be in mild respiratory distress, and is unable to stop rapid, panting breathing.

2. Subject often takes loud, rapid and shallow breaths. Subject appears to be in mild respiratory distress, and is unable to stop rapid, panting breathing except when her attention is held elsewhere.
3. Subject sometimes takes loud, rapid and shallow breaths. Subject does not appear to be in respiratory distress and is unable to stop rapid, panting breathing when her attention is held elsewhere.

**Moaning/Shouting**

1. Subject often vocalises loudly and without intent. Subject appears in distress, and cries, shouts or moans indiscriminately.
2. Subject sometimes vocalises loudly and without intent. Subject appears in mild distress, and cries, shouts or moans indiscriminately.
3. Subject rarely vocalises loudly and without intent. Subject does not appear to be in distress, only occasionally cries, shouts or moans indiscriminately.

**Tongue Chewing**

1. Subject consistently and repeatedly chews her tongue in an uncontrollable and stereotypical manner. Action interferes with feeding or vocalising.
2. Subject often repeatedly chews her tongue in an uncontrollable and stereotypical manner. Subject is able to stop tongue chewing when feeding or vocalising.
3. Subject occasionally chews her tongue in an uncontrollable and stereotypical manner. Subject is able to stop tongue chewing when feeding or vocalising.

**Vacant Staring**

1. Subject shows no or little interest in her surroundings. Subject constantly stares at nothing in particular and pays no attention to any visual stimuli.
2. Subject shows little interest in her surroundings. Subject often stares at nothing in particular and pays little attention to any visual stimuli.
3. Subject shows some interest in her surroundings. Subject sometimes stares at nothing in particular but pays some attention to visual stimuli.

**Bruxism**

1. Subject constantly and consistently grinds her teeth and moves her jaw in a stereotypical and repeated manner. Action interferes with feeding and is highly associated with “biting” and “moaning”.
2. Subject often grinds her teeth and moves her jaw in a stereotypical and repeated manner. Action does not interfere with feeding and is associated with “biting” and “moaning”.
3. Subject rarely grinds her teeth and moves her jaw in a stereotypical and repeated manner. Action does not interfere with feeding and is not associated with “biting” or “moaning”.

**Breathholding/Apnea**

1. Subject goes through constant cycles of holding her breath for prolonged periods of time. Highly associate with “superficial breathing” and “valsalva manouver”. Subject appears in moderate respiratory distress.
2. Subject goes through occasional cycles of holding her breath for moderate periods of time. Highly associate with “superficial breathing” and “valsalva manouver”. Subject does not appear in respiratory distress.
3. Subject rarely holds her breath for moderate periods of time. Subject does not appear in respiratory distress.

**Valsalva Manouver**

1. Subject constantly holds her breath and then forcibly expels it in a stereotypical manner. Subject is unable to stop performing the valsalva manouver and appears to be in moderate distress.
2. Subject often holds her breath and then forcibly expels it in a stereotypical manner. Subject has difficulty stopping performing the valsalva manouver and appears to be in mild distress.
3. Subject sometimes holds her breath and then forcibly expels it in a stereotypical manner. Subject does not have difficulty stopping performing the valsalva manouver and does not appear to be in distress.

**Pointing**

1. Subject has extreme difficulty gesturing toward any stimuli of interest to her using her body or hands.
2. Subject is able, on occasion, to indicate toward any stimuli of interest to her using her body or hands.
3. Subject consistently and repeatedly attempts or succeeds in gesturing toward a stimulus of interest using her body or hands.

**Manipulating**

1. Subject is completely incapable or can barely hold objects of interest and is unable to feed herself without aid.
2. Subject can hold objects without aid but is unable to turn them around or operate them appropriately in any way.
3. Subject can feed herself relatively easily and can turn and play with objects of interest

**Reaching for something**

1. Subject is completely r poorly incapable of reaching for objects of interest or of indicating her interest in these objects in any way. Highly associated with “pointing” and “manipulating”
2. Subject shows limited ability to reach for and hold objects of interest.
3. Subject can reach for and hold objects of interest with relative ease.

**Waving Goodbye**

1. Subject is incapable of using hand gestures to communicate goodbye, or anything else, to any other person.
2. Subject attempts to use hand gestures to communicate goodbye, or anything else, to any other person.
3. Subject tries to and consistently succeeds in using hand gestures to wave goodbye or communicate in any other way with other persons.

**Yes/No with head movement**

1. Subject is incapable of using head gestures to communicate yes or no, to any other person.
2. Subject attempts to use head gestures to communicate yes or no, to any other person.
3. Subject tries to and often succeeds in using head gestures to communicate yes or no to other persons.

**Reactivity to a Call**

1. Subject displays extremely poor or no outward indication of attention on being called by another person.
2. Subject turns toward a call made by another person, but has no other reaction apart from this.
3. Subject turns and reacts to the person calling, and communicating in a limited sense with the person who has called them.

**Smiling in response to a stimulus**

1. Subject does not react to any stimulus of interest (Eg, a video/toy).
2. Subject cries or gives some other inappropriate response to a stimulus of interest.
3. Subject smiles and laughs appropriately toward a stimulus of interest to some extent.

**Deliberate Vocalisation**

1. Subject rarely cries and laughs at appropriate times toward appropriate stimuli.
2. Subject indicates by deliberate vocalisation her emotions toward a particular stimulus.
3. Subject attempts to use her vocalisations to communicate to some degree with another person.

**Attention**

1. Subject shows very little interest in her surroundings. Subject constantly stares at nothing in particular and pays very little attention to any stimuli.
2. Subject shows little interest in her surroundings. Subject sometimes pays limited attention to a stimulus of interest.
3. Subject shows interest in her surroundings. Subject pays attention to a stimulus of interest.
